# Pentraxin 3(PTX 3): An Endogenous Modulator of the Inflammatory Response

**DOI:** 10.1155/2012/920517

**Published:** 2012-04-11

**Authors:** P. Kunes, Z. Holubcova, M. Kolackova, J. Krejsek

**Affiliations:** ^1^Department of Cardiac Surgery, University Hospital and Medical School of Hradec Kralove, Charles University in Prague, 12843 Prague, Czech Republic; ^2^Department of Clinical Immunology and Allergology, University Hospital and Medical School of Hradec Kralove, Charles University in Prague, 12843 Prague, Czech Republic

## Abstract

Inflammatory or anti-inflammatory? That is the question as far as the acute-phase response and its mediators, the pentraxins, are concerned. Only some ten years ago, the classical or short pentraxin C-reactive protein and the newly discovered long pentraxin PTX3 were considered to exert most of the detrimental effects of acute inflammation, whether microbial or sterile in origin. However, accumulating evidence suggests an at least dichotomous, context-dependent outcome attributable to the pentraxins, if not a straightforward anti-inflammatory nature of the acute-phase response. This paper is focused on the inherent effects of pentraxin 3 in inflammatory responses, mainly in coronary artery disease and in *Aspergillus fumigatus* infection. Both are examples of inflammatory reactions in which PTX3 is substantially involved; the former sterile, the latter infectious in origin. Apart from different inducing noxae, similarities in the pathogenesis of the two are striking. All the same, the introductory question still persists: is the ultimate impact of PTX3 in these conditions inflammatory or anti-inflammatory, paradoxical as the latter might appear? We try to provide an answer such as it emerges in the light of recent findings.

## 1. Danger Recognition

The innate immune system provides the first line of defense against microorganisms with which humans are daily contacted. The encounter with the microbial world occurs either on the body's external surface, that is, on the skin, or on its internal (mucosal) surfaces in the airways and in the gastrointestinal tract. The vast majority of these steady-contact microbes are not inherently pathogenic. Nevertheless, their eventual threat to the host may vary considerably according to both external/environmental and to internal conditions, the latter being reflected by the ability of the host to mount an appropriate immune response. Whenever needed, this response must go on to involve the adaptive immune reaction(s). Basically, the same holds true for virtually pathogenic microorganisms. An infection may be brought right under control if confronted with an immunologically competent host. Actually, the net result of pathogen-host interaction, that is, whether health is maintained or disease develops, relies on the balance between pathogen virulence and the capacity of the individual immune response. The innate and adaptive immune reactions form a continuum of closely interrelated steps with innumerable positive and negative feedback loops. Therefore, they should be viewed as two sides of the same coin [1].

To initiate a defense reaction, first of all, a microorganism must be sensed and recognized by the immune system as potentially harmful. It has been established that microorganisms are not recognized in their individual complexity. Instead, several sets of highly conserved molecular moieties which are shared by large groups of microorganisms are implicated in the process of being recognized. These molecular motifs, collectively referred to as *alarmins *or *pathogen-associated molecular patterns *(PAMPs), are committed to elicit a *danger signal *within the immune system. Importantly, closely resembling or even identical molecular structures are present in the host's own cells. Under normal conditions, these molecules are well hidden inside the cellular cytoplasm, far beyond the reach of the recognition machinery. In cells undergoing apoptotic or necrotic death, the hallmark of sepsis or ischemia-reperfusion injury, PAMPs/alarmins are promptly exposed on the surface membrane of the host's cells or even released into the extracellular space [2, 3]. Another way to exhibit own PAMPs/alarmins, albeit in a slow, gradual process, occurs in low-grade inflammation. Examples thereof are atherogenesis or rheumatoid arthritis. Inflammatory diseases which develop in the absence of microorganisms are called *sterile inflammation *[4]. 

The constituents of the immune system which are involved in the process of recognizing PAMPs, either of microbial origin in bacteremia/sepsis or modified self-structures in sterile inflammation, are represented by germ line-encoded receptors known as *pattern recognition molecules *(PRMs). The occurrence of PRMs is not confined to canonical components of the immune system, but other cells are also involved. From the viewpoint of cardiology/cardiac surgery, endothelial cells play a prominent role [5].

Within the human body, pattern recognition molecules/receptors are present either as *cell-associated- *or as *fluid-phase molecules.* The former are localized in most tissues, whereas the latter are distributed in the liquid compartment, that is, mainly, but not exclusively, in the blood. Cell-associated receptors are made up by endocytic/scavenger receptors, signalling receptors (e.g., toll-like receptors), and nucleotide-binding oligomerization domain- (NOD-)like receptors [6]. The fluid-phase molecules represent evolutionary ancestors of antigen-specific antibodies. This heterogeneous group of molecules consists of three clearly defined subgroups, that is, the collectins, the ficolins, and the pentraxins. All of them are implicated in complement activation, pathogen opsonisation, and/or self versus modified-self versus non-self discrimination [7].

## 2. A Brief Glance at the Pentraxin Superfamily

The pentraxins form a superfamily of multifunctional proteins which have been conserved in phylogeny from arachnids to mammals. The pentraxin superfamily is distinguished by the presence in their C(carboxy)-terminal region of a ~200 amino acid domain containing a highly conserved motif of 8-amino-acid sequence, which has been named the *pentraxin signature *(HxCxS/TWxS, where x is any amino acid). Based on the primary structure of the protomer, the pentraxins branch off into two groups: the short constituents and their long counterparts [8].

Short pentraxins are about 25-kDa proteins sharing a common structural organization of five or ten subunits that are assembled in pentameric radial symmetry. The classical short pentraxins C-reactive protein (CRP) and serum amyloid P component (SAP) are acute-phase proteins in humans and in mice, respectively. They are manufactured in the liver under the guidance of inflammatory cytokines, most prominently of interleukin (IL)-6 and, to a lesser degree, of IL-1*β*. CRP is the major acute-phase reactant in humans. Its levels are scarcely detectable in plasma of healthy persons, in whom CRP concentrations do not exceed 3 mg/L (3 *μ*g/mL). However, CRP plasma concentrations increase as much as 1000-fold under inflammatory conditions, irrespective whether of bacterial (sepsis) or sterile (sepsis-like) origin. Classically, the latter develops in patients after trauma and surgical operations or in patients with acute coronary syndromes (unstable angina pectoris/acute myocardial infarction). CRP and SAP bind their respective ligands in a calcium-dependent manner. Both of them play important roles in innate resistance to microbes and in scavenging pathogenic cellular debris including extracellular matrix components [9]. Pentraxin 3(PTX3) was identified in the early 1990s in human endothelial cells and fibroblasts as a TNF-*α*- or IL-1*β*-inducible mRNA and protein, respectively. IL-6 does not deal with PTX3 production. Despite manifest similarities of action, PTX3 differs from its short associates in many basic aspects, such as gene organization, chromosomal localization, cellular sources, inducing stimuli and the recognized ligands [10].

## 3. Genetic Aspects of Pentraxin 3

The human *ptx3 *gene is localized on the chromosome 3q band 25. It is composed of three exons, the first two of which encode for the signal peptide and the N-terminal domain (amino acids 18–179), respectively. The third exon encodes for the C-terminal domain featuring the pentraxin signature (amino acids 179–381) [11]. Immature myeloid dendritic cells were supposed to be the prevailing cellular population capable to produce PTX3. However, stimulus-induced PTX3 has been detected in other cellular populations, such as the monocytes/macrophages, smooth muscle cells, kidney epithelial cells, synovial cells, chondrocytes, adipocytes, alveolar epithelial cells, glial cells, fibroblasts, and, of course, in endothelial cells [12]. PTX3 promoters contain enhancer-binding elements which, during proteosynthesis, fine-tune final impact of PTX3 on its target structures. The most prominent ones are activator protein-1 (AP-1), nuclear factor-kappa B (NF-*κ*B), and selective promoter factor 1 (SP1) [13]. Briefly, AP-1 enhances basal transcription of PTX3, whereas the NF-*κ*B binding site is operative in the response to inflammatory cytokines TNF-*α* and/or IL-1*β*. Tissue-specifically, the above-mentioned transcription factors are complemented, in their proteosynthesis-modulating activities, by enzymatic biochemical pathways. *In lung epithelial cells* challenging acute inflammation, PTX3 mRNA as such is induced by TNF-*α*; nevertheless, PTX3 protein generation itself does not require consequent NF-*κ*B transcription. Instead, PTX3 is manufactured by way of the c-Jun N-terminal kinase pathway [14]. *In endothelial cells*, expression of PTX3 is readily induced by TNF-*α* and IL-1*β*. Thereafter, an acute cellular alteration sets in, in which the endothelial cell is converted from a quiescent, anti-inflammatory phenotype, to a procoagulant and proinflammatory cellular surface. This is the case in sepsis/infection or in acute myocardial infarction. On the other hand, endothelial cells are implicated in low-grade sterile inflammation that underlies atherosclerosis and small vessel vasculitides. A counter-regulatory pathway which abrogates unwanted inflammation is carried out by high-density lipoprotein 3 (HDL3) subfraction. This HDL3-controled PTX3 production evades classical regulatory mechanisms in that it relies on the activation of the PI3K/Akt pathway via G-coupled lysosphingolipid receptors. The lysosphingolipid receptor S1P, which is the one responsible for this alternative PTX3 synthesis, is a protein particle carried by HDL3. In human endothelial cells, therefore, PTX3 accomplishes dual outcome according to the respective activators: (i) an inflammatory effect induced by TNF-*α* and/or IL-1*β* or (ii) an anti-inflammatory effect induced by S1P/HDL3. The latter is further translated into increased NO-dependent vasorelaxation, endothelial cell antiapoptotic effects, and increased TGF-*β* expression with ensuing atheroprotective modulation [15]. PTX3 generation is also regulated in a cell-dependent manner by glucocorticoid hormones (GHs). GHs support the production of PTX3 in *fibroblasts *and *endothelial cells*, whereas they suppress PTX3 production by haematopoietic cells (*dendritic cells *and *macrophages*). Accordingly, intravenous administration of a glucocorticoid hormone increases blood levels of PTX3 [16].

## 4. Pentraxin 3 Storage in Neutrophils

Apart from the generation of PTX3 in a response to inflammatory stimuli, there is also a constitutive, ready-made form of PTX3 which is stored in specific (lactoferrin^+^) granules of neutrophils. After these cells have been activated by appropriate stimuli, this preformed amount of PTX3 is released into the extracellular space. This is a virtually mature, that is, glycosylated form of monomeric PTX3 which, once occurring extracellularly, assembles into multimers of five subunits. The PTX3 glycosidic moiety presents a complex of bi-, tri-, and tetra-antennary sialylated structures, the relative number of which undergoes changes reflecting the inflammatory conditions in which actual PTX3 protein has been generated. Glycosylation of PTX3 seemingly functions as a fine-tuning mechanism of the mature protein. Thus, neutrophils represent a ready-to-use reservoir of PTX3 guaranteeing its early release and early activity in acute inflammation [17]. Under normal steady-state conditions, circulating neutrophils exhibit a short half-life terminated by apoptotic death, which serves to protect the host from any undue damage inflicted by these cells. Whenever inflammation is evoked, those stimuli which account for early PTX3 release delay concomitantly neutrophil apoptotic death. This is a natural feedback mechanism maintaining neutrophil numbers within required limits; otherwise, the host would incur premature neutrophil exhaustion leading to attenuation of inflammation. Delayed neutrophil apoptosis is not a selective mechanism. Prolonged neutrophil life-span is needed to support the host protection against infection/sepsis. However, the same scenario, albeit occurring in a slow step-by-step manner, is set in motion in chronic sterile inflammation in which increased numbers of activated neutrophils are harmful to the host. Atherosclerosis may serve as an example thereof, although participation of neutrophils in this process was long underestimated [18].

Upon inflammatory activation, neutrophils release about 25% of their actual PTX3 content. Part of the extruded protein remains associated with the parent cell by way of neutrophil extracellular traps (NETs). This is a chromatin material assembled with nuclear proteins which has been set free concomitantly with PTX. Localization of PTX3 in neutrophil extracellular traps contributes to the generation of an antimicrobial microenvironment which augments local capacity to trap and kill microbes. Given the abundance of neutrophils both in the circulation and in the inflammatory foci, these cells whose life-span has been prolonged into the bargain constitute the main source of PTX3 right after the onset of infection or in acute sterile inflammation, such as myocardial infarction. Neutrophil-released PTX3 is functionally competent well before *de novo *synthesis by other cells delivers supplemental amounts of the protein. At the same time, neutrophil-derived PTX3 accounts for another feedback mechanism which restricts excess transmigration of activated neutrophils into the host's tissues, thus dampening unwanted dissemination of inflammatory reactions [19].

## 5. Regulation of Pentraxin 3 Production

Activation stimuli which control PTX3 synthesis and release can be enumerated as follows: (i) proinflammatory cytokines (IL-1*β*, TNF-*α*), (ii) TLR agonists, namely lipopolysaccharide (LPS), (iii) distinct microbial moieties (OmpA, lipoarabinomannans), and/or (iv) some intact microorganisms. Taking into account the complexity of the inflammatory/immune reactions, more than one stimulus is usually employed to set off PTX3 production. To initiate the immune response, cell-expressed receptors tightly cooperate with their fluid-phase counterparts after they have recognized hostile microorganisms (*non-self*) via the latter's PAMPs. Essentially, the same scenario proceeds in cases of sterile inflammation, after the recognition machinery has discriminated own modified or damaged cell structures (*altered self*) [20]. To give an example, macrophage scavenger receptor CD36 mediates binding and internalization of Gram-positive bacteria by cooperating with toll-like receptors TLR2 and TLR6, thereafter initiating production of proinflammatory cytokines TNF-*α*, IL-1*β*, IFN-*γ*, and many others. Concurrently, production of both short and long pentraxins CRP and PTX3 by their paternal cells may be started off. In contrast to the current paradigm which claims that cytokines considered proinflammatory should be activated to start the inflammatory/innate immunity response, IFN-*γ* downregulates PTX3 production in dendritic cells and in monocyte-macrophage cell lines. To achieve this, IFN-*γ* decreases PTX3 mRNA transcription and reduces PTX3 transcript stability, respectively [21]. On top of it, LPS-induced PTX3 expression is downregulated by (i) interleukin-4, (ii) 1*α*,25-dihydroxivitamin D3, and (iii) prostaglandin E2 [22]. Additionally, PTX3 by itself does not bind to (i) lipopolysaccharide (LPS), (ii) lipoteichoic acid (LTA), (iii) enterotoxins A, and B, (iv) exotoxin A and (v) N-acethylmuramyl-L-alanyl-D-isoglutamine (MDP). Interleukin-(IL-)10, a most prominent anti-inflammatory cytokine, enhances LPS-induced PTX3 production, however, paradoxically it might appear. Due to the impact of IL-10 on dampening inflammation and supporting wound healing, this fact implies that PTX3/IL-10 would play an imortant role in tissue repair [23].

## 6. Pentraxin 3 in Cardiovascular Diseases

Important pieces of knowledge regarding PTX3 behavior in cardiovascular diseases, such as atherosclerosis and acute myocardial infarction (AMI), have been gained in the last ten years. As early as in 2000, Peri and coworkers published in *Circulation *their results from a cohort of 37 patients who had presented with an AMI. The authors showed that in these patients, plasma levels of PTX3 peaked as early as ~6 hours after the onset of chest pain. Thus, maximal concentration of PTX3 was available much earlier than that of CRP which is achieved after ~48 hours. In AMI patients, individual levels of PTX3 were attained independently of the extent of myocardial necrosis or incident heart failure assessed by the Killip class [24]. Four years later, these authors extended their original findings by adding that in AMI patients, PTX3 supplied the most powerful information regarding three-month mortality after the index event. In their study, prognostic value of PTX3 was superior to that provided by CRP, CK, TnT, and NT-proBNP [25]. Additional studies introduced PTX3 as a prognostic biomarker in heart failure, both acute and chronic, which was unrelated to an AMI [26, 27]. In the cardiovascular health study, PTX3 has been established to associate with the incidence of CAD and all-cause mortality in CAD patients, independently of CRP and other classical risk factors [28]. In our own study dealing with plasma PTX3 changes in patients undergoing coronary artery bypass grafting, we expectedly documented hundred-fold elevations of CRP in all patients, none of whom suffered any peri- or postoperative complication. However, PTX3 did not exceed the level of 2 ng/mL in any blood sample collected during or up to the 7th day after the operation, this respective value has been set as normal for healthy humans [29]. Studies examining the metabolic syndrome, a prominent risk factor of CAD, do also point out to discordant comportment of CRP and PTX3. Whereas CRP appears to correlate *positively* with (i) body weight, (ii) body-mass index, (iii) waist circumference, (iv) fasting plasma glucose, and (v) plasma IL-6 levels and *negatively* with (i) HDL cholesterol and (ii) adiponectin, PTX3 displays the opposite trends. It can be soundly assumed that the functions of PTX3 and CRP complement or even overlap in some situations, wheres in others they diverge substantially. There are even first hints that PTX3 might play an as yet unrecognized protective role in atherosclerosis and its complications [30].

## 7. Pentraxin 3 in Atherosclerosis

Studies examining PTX3 in the process of atherogenesis found this long pentraxin to be expressed in human vascular smooth muscle cells via atherogenic lipoproteins [31], to up-regulate tissue factor expression both in human endothelial cells [32] and in activated monocytes [33], and to occur extensively in advanced atherosclerotic plaques. Therein, the source of PTX3 is not confined to macrophages and surviving endothelial cells [34], but, importantly, PTX3 is also present in infiltrating neutrophils [35], a cell population increasingly recognized as being active in the initial phase of the disease as well as in its complications [36, 37]. Clinically, PTX3 has been found to operate actively in unstable angina pectoris [38, 39] and in restenosis after coronary artery stent introduction; however, without identifying its source(s) in these conditions [40]. Furthermore, PTX3 displays a predilection to segments of vascular or synovial inflammation in rheumatic diseases, the pathogenesis of which is supposed to have much in common with that of atherosclerosis [41]. As it is, the inaugural question stands out with an intriguing acuity: is pentraxin 3 an inflammatory/harmful or an anti-inflammatory/benefitial mediator?

This question has been partly resolved with the accession of genetically modified mice in whom the PTX3 gene has been silenced (*ptx*3^−/−^ mice). These animals do not produce any traces of PTX3 protein. When cross-bred with apolipoprotein E gene-deficient (*apoe* ^−/−^) mice, which is a murine phenotype closely recapitulating the development of human atherosclerosis, mouse strains which result from these combinations (PTX3^+/+^ or PTX3^+/−^ ApoE^−/−^ versus PTX3^−/−^ ApoE^−/−^) yielded exciting insights into the involvement of PTX3 in atherogenesis. During the course of aging, PTX3^−/−^ apoE^−/−^ mice develop, in line with expectations, increasing atherosclerosis as a function of time. When fed Western-type diet, these mice display an even increased extent of atherosclerosis. In both settings, that is, normal chow versus Western-diet-fed mice, PTX3 amounts correlated positively with the atherosclerotic burden. By contrast, in PTX3^+/+^ apoE^−/−^ double knockout mice, the atherosclerotic affliction is unexpectedly enhanced instead of being diminished, which should have been the case if PTX3 was an unequivocal atherogenic mediator. Further studies specified that in atherosclerosis-prone mice, total absence of PTX3 was pronounced in the vascular wall by increased upregulation of (i) proinflammatory mediators such as the chemokines (e.g., CCL2, CCL3, CCL5, CCL7, CCL8) or cytokines (e.g., IL-4, IL-6, TNF-*α*), (ii) transcription factors such as NF-*κ*B and the related protein Irak1, Fos, Jun, GATA3, GATA4, Egr2, Egr3, (iii) key mediators of the inflammatory response (e.g., TLR-2, TLR-4), and/or (iv) adhesion molecules responsible for the onset of endothelial dysfunction: intercellular adhesion molecule-1 (ICAM-1), vascular cell adhesion molecule-1 (VCAM-1), endothelial leukocyte adhesion molecule-1 (E-selectin), platelet/endothelial cell adhesion molecule (PECAM) [42].

## 8. Pentraxin 3 in Myocardial Infarction and in Ischemia/Reperfusion Injury

Alberto Mantovani's team furthermore evidenced that in genetically modified mice, PTX3 plays a protective role in acute myocardial infarction, notably in the reperfusion phase. It must be pointed out here that PTX3 is not expressed in the heart under resting conditions, either murine or human. During reperfusion of a previously ischemic myocardium (or, practically, any tissue), an inflammatory response is elicited by explosive production of reactive oxygen species. In the course of reperfusion, an injury develops which is characterized by leukocyte activation and recruitment thereof into the reperfused area; by endothelial dysfunction which paves the way to these cells'apoptotic or necrotic death and/or by cardiomyocyte cell death which occurs as a result of blood flow defects (the “no-reflow” phenomenon). In a model of coronary artery ligation/reperfusion, PTX3^−/−^ mice exhibit more extensive myocardial damage compared to their wild-type littermates. Specifically, reperfusion injury is manifest as (i) increased activated neutrophil and monocyte/macrophage infiltration to the damaged tissue, (ii) decreased number of tissue capillaries including extension of the no-reflow area by endothelial swelling which results in an obstruction of the remaining vessels, and/or (iii) increased number of apoptotic cardiomyocytes caused either by microcirculatory drop-outs, or inflicted to the cardiomyocytes by activated complement. Conversely, administration of exogenous PTX3 to PTX3-deficient mice dampens this injury down to the level experienced by their wild-type littermates. Administration of surplus PTX3 protein seems to confer a supplemental protection not only to the deficient, but also to normal, that is, PTX3^+/+^ mice [43]. As far as humans are concerned, the situation requires much of further research.

## 9. Interaction of Pentraxin 3 with Platelets in Acute Myocardial Infarction

Activated platelets cause much of tissue damage in myocardial infarction. Hence, they are the predominant target cells of PTX3. Upon extrusion into the extracellular space, neutrophil-originating PTX3 binds to adjacent platelets which, thereupon, lose much of their robust inflammatory armamentarium. PTX3-stained platelets are thereafter resistant to the formation of platelet-platelet homoaggregates or to platelet-neutrophil or platelet-monocyte heteroaggregates. Furthermore, PTX3 broadly impacts the adhesion molecule P-selectin, which is known to play distinct roles in atherogenesis. PTX3 brings about downregulation of P-selectin-dependent neutrophil recruitment to inflammatory sites and of P-selectin-induced cellular heteroaggregate formation. Hence, decreased numbers of microaggregates are present that would impair blood flow in the microcirculation, not to speak of their additional noxious potential. In acute coronary syndromes, the overall procoagulant state is increased, including excess tissue factor formation by different cell types which makes the blood more “sticky” [44].

Intervention of either endogenous or exogenous PTX3 mitigates the development of the “no-reflow” fenomenon, that is, sustained tissue hypoxia/anoxia despite successful recanalization of large arteries. The jeopardized tissue is imbibed, irrespective of its extent, by a dense infiltrate which is composed largely of granulocytes and macrophages. The same applies to the early necrotic scar before its fibrotic transformation. PTX3 can be detected in most of the infiltrating macrophages and remaining endothelial cells, whereas neutrophils are scarcely found by this time point. If so, a few granulocytes are localized mainly in the center of the lesion, whereas in the outer parts, PTX3 is found only extracellularly. Cardiomyocytes are constantly devoid of PTX3 [45]. On the subcellular level, activities of PTX3 impact the transcription factor NF-*κ*B and its modifying effects on proteosynthesis, since the beneficial outcome is lost in IL-1RI- or MyD88-deficient mice. MyD88 is a cellular adaptor protein downstream of the receptors for TLR and IL-1R. MyD88- or IL-1R-deficient mice are unable to upregulate PTX3 expression and in cases of acute coronary events, these artificial animals behave like those who are entirely lacking the long pentraxin (PTX3-deficient or PTX3^−/−^ mice). Thus, the IL-1R-MyD88 pathway plays a dominant role in ptx3 mRNA induction in heart ischemia. In MyD88- or IL-1R-deficient mice, ample PTX3 protein may be available, but PTX3-borne signal cannot be transmitted from the surface membrane of the target cell to its nucleus where proteosynthesis controled by NF-*κ*B takes place, including that which should supply, among other mediators, more amounts of PTX3 protein [46].

*In vitro, *preincubation of apoptotic cells with PTX3 enhances complement factor C1q binding and factor C3 deposition on the cellular surface, thus mimicking participation of PTX3 in complement-mediated clearance of apoptotic cells. The *in vivo *activity of PTX3, however, is dichotomous, depending upon whether complement has been activated *systemically* in circulating blood or *locally* in the inflammatory focus. Furthermore, PTX3 binds to late apoptotic neutrophils and inhibits their phagocytosis by monocyte-derived macrophages. Consequently, PTX3^−/−^ mice display increased C3 deposition in their ischemic myocardium, whereas exogenous PTX3 reduces in them complement activation, provided, of course, that the IL-1R-MyD88 pathway is intact. *In vitro, *inhibition of complement by fluid-phase PTX3 occurs at concentrations in the same range as those achieved in PTX3^−/−^ mice 24 hours after myocardial reperfusion. Therefore, it can be expected that PTX3 protective activities in later phases of an AMI result from its impact on the complement cascade, which accounts for the salvage of apoptosis-destined, but still viable cells [47].

## 10. Pentraxin 3 Interaction with the Complement System

PTX3 is composed of a unique N-terminal region coupled to a C-terminal domain, which is homologous to the short pentraxins CRP and SAP. The N-terminal region of PTX3 is unrelated to any protein structure known so far. Studies carried out with recombinant preparations of the N- and C-terminal domains of PTX3, respectively, indicate that the N-terminal region binds ligands such as (i) fibroblast growth factor-2 (FGF2), (ii) inter-*α*-inhibitor, and/or (iii) conidia of *A. fumigatus*; whereas the pentraxin-like C-terminal domain of PTX3 interacts with the complement factor C1q and the adhesion molecule P-selectin. On the other hand, both the N- and the C-terminal domains have been implicated in the interaction with the complement factor H [48].

The impact of PTX3 on complement activation is a complex issue. PTX3 binds C1q, the first component of the classical complement cascade, by interacting with the C1q globular head (gC1q) and, in particular with charged residues on the apex part of the molecule which encompass all three C1q chains (i.e., gC1qA, gC1qB, and gC1qC) [49]. PTX3 binds to *immobilized *C1q, such as is aggregated on the surface of bacterial walls. Interaction of PTX3 with surface-bound C1q leads to the *activation *of the classical pathway, as can be assessed by the deposition of C3 and C4 fragments on the target cells. PTX3 itself, however, does not interact either with C3 or with C4. In contrast, the presence of PTX3 in circulating blood leads to a dose-dependent inhibition of C1q hemolytic activity, an event in which PTX3 limits C1q binding to antibody-sensitized erythrocytes. Hence, binding of fluid-phase PTX3 to *free *C1q results in the *inhibition* of the classical cascade of complement. As it is, PTX3 exerts a dual role on the classical pathway of complement: it supports clearance of corpuscular material that binds PTX3, such as microbes or apoptotic cells, while it restricts unwanted complement activation in circulating blood [50].

The short pentraxin CRP, in addition to the classical pathway, modulates the *alternative pathway *of complement via interaction with factor H, the main soluble regulator of the alternative pathway. PTX3 can also interact with factor H, thus leading to increased factor H and factor iC3b deposition on apoptotic cells. Factor iC3b is the prevalent opsonin generated during complement activation, which performs an important activity in the process of complement-mediated phagocytosis. The net result of PTX3 impact on the alternative pathway of complement is mirrored by increased factor H deposition on PTX3-coated surfaces, that is, activation of complement by the alternative pathway in response to appropriate stimuli is increased. At the same time, however, exaggerated complement activation by the alternative pathway, which is active even under steady-state conditions to guarantee an immediate-early weapon in cases of sudden bacterial intrusion and which might, in a pathogen-free environment, inflict an erroneous damage to the host, is being held under control [51].

PTX3 interacts also with components of the lectin pathway. The mannose binding lectin (MBL) binds PTX3 via its collagen-like domain. MBL/PTX3 complexes recruit C1q and elicit C3 and C4 deposition on target cell surfaces, such as those of *Candida albicans*. Phagocytosis thereof is mightily enhanced. Furthermore, pentraxin 3 interacts with another fluid-phase PRM, namely, L-Ficolin (alternatively known as ficolin-2). Of note, ficolin-2 has been shown to bind conidia of *Aspergillus fumigatus. *This ficolin 2-*A.fumigatus* interaction is supported by PTX3 and conversely, PTX3-*A.fumigatus* binding is supported by ficolin-2. PTX3 also promotes ficolin-2-induced complement deposition on the surface of *A. fumigatus*, thus aiding the host to combat the infection [52]. Recently, an interaction between M-ficolin (ficolin-1) and PTX3 has been described in which immobilized PTX3 triggers M-ficolin- induced activation of the lectin complement pathway [53].

## 11. Pentraxin 3 Interaction with Microbial Moieties

Opsonization of target cells (invading pathogens or altered self structures) results in (i) facilitated pathogen recognition (and increased phagocytosis and killing) and in (ii) *innate* immune cell activation (and increased inflammatory cytokine and nitric oxide production). Moreover, opsonization by PTX3 is evidently linked to the activation of an appropriate *adaptive* immune response (i.e., dendritic cell maturation and polarization). PTX3 opsonizes manifold pathogens or pathogen-derived ligands, most importantly zymosan, *Paracoccidioides brasiliensis *or conidia of *Aspergillus fumigatus *[54]. Other interactions concern Gram-positive and Gram-negative bacteria, namely, *Staphyloccocus aureus*, *Pseudomonas aeruginosa*, *Salmonella typhimurium, Streptococcus pneumoniae, *and* Neisseria meningitidis. *Even viruses are affected, namely, human and murine cytomegalovirus (CMV) or H3N2 influenza virus.

PTX3 binds the outer membrane protein A from *Klebsiella pneumoniae *(KpOmpA). OmpA, an archetypical pathogen-associated molecular pattern, is a prominent membrane component of Gram-negative bacteria, which has been conserved in phylogeny among the *enterobacteriaceae *family. As such, KpOmpA is recognized by monocytes and dendritic cells (DCs) via two members of the scavenger receptor family (the *lectin-like oxidized low-density lipoprotein receptor-1 *or LOX-1 and the *scavenger receptor expressed by endothelial cell-I *or SREC-I) and is able to induce a TLR2-dependent proinflammatory response [55]. This inflammatory response includes supplemental PTX3 production via a positive feedback loop which, in its turn, promotes the original inflammatory response brought about by KpOmpA, in terms of inflammatory cell recruitment and inflammatory cytokine release. Due to PTX3 participation, this response is complement modified [56]. In murine *Klebsiella pneumoniae *infection, the difference between death and recovery relies on PTX3-evoked fine-tuning of the balance between tumor necrosis factor (TNF-*α*) and nitric oxide (NO) production. By (i) shifting the balance of these antagonistic mediators in one or another way and (ii) depending upon the inaugural inflammatory response which must deal either with a *high* or a *low* bacterial inoculum, PTX3 will either favor or disfavor the ability of the host to combat the infection [57]. Essentially, these principles hold true for any inflammation, be it of microbial or sterile origin. Examples thereof will be presented followingly.

## 12. Pentraxin 3 and Immune Responses in *Aspergillus Fumigatus* Infection

*Aspergillus fumigatus *is an ubiquitary fungus which elicits a broad spectrum of diseases in immunocompromised or atopic subjects, ranging from severe pulmonary infections to allergy. Aspergillosis is a major life-threatening infection in patients with *defective phagocytosis*, the hallmark of chemotherapy- or radiotherapy-induced neutropenia and monocytopenia [58]. From among inborn immunodeficiencies, *chronic granulomatous disease *(CGD) is of interest in this context. Pathophysiologically, this condition displays defective superoxide production by the enzyme NADPH. The clinical course is characterized by increased susceptibility to infections. Opportunistic pathogens such as *Aspergillus fumigatus* are involved. Prophylaxis with trimethoprim-sulfamethoxazole, itraconazole, and, in selected cases, administration of IFN-*γ* may be efficient, but often imperfect. Excessive inflammation is treated with systemic corticosteroids which, however, further suppress the patient's immunity. Gene-replacement therapy for patients lacking a suitable stem cell donor is still an experimental procedure. Neutrophils, although reduced in numbers and/or in function, are of paramount importance in the institution of a protective inflammatory response. Inflammation by itself may confer some protection. Notwithstanding, tight control of defense reactions is a vital condition. Unleashed inflammation is deleterious. On top of these problems, CGD patients may develop autoimmune diseases, a condition which mirrors generation of neoantigenic determinants, presumably by reactive oxygen and nitrogen species, which is the more so astonishing that the NADPH enzyme is functionally deficient [59].

In mice, genetic loss-of-function studies have yielded results that might be, at least in part, applied to humans. First, PTX3-knockout mice (*ptx*3^−/−^), in whom the gene encoding for PTX3 has been silenced, are highly susceptible to invasive pulmonary aspergillosis. The course of the disease recapitulates defective recognition and killing of *aspergillus *conidia by PTX3-deficient neutrophils, dendritic cells, and alveolar macrophages [60]. Second, the p47phox^−/−^ mice closely mimic human CGD, most importantly the defective, albeit detrimental inflammation. Once these mice have been infected with *Aspergillus fumigatus, *their own production of PTX3 is both delayed and insufficient. Administration of exogenous PTX3 in early infection is able to rescue antifungal resistance and, at the same time, to restrain the exaggerated inflammatory response to the fungus by way of curbing the IL-23/Th17 inflammatory axis [61]. This beneficial reversal is produced via downregulation of IL-23 production by dendritic cells and epithelial cells, an effect associated with *limited *expansion of IL-23R^+^ *γδ* ^+^ T cells. These cells are important producers of IL-17A, a mighty inflammatory cytokine and a neutrophil chemoattractant which is involved in many inflammatory diseases, once again either microbial (e.g., *Borrellia burgdorferi, Mycobacterium tuberculosis*) or sterile (atherosclerosis including its complications) in origin. Consequently to IL-17A decrease, protective Th1/T_reg_ responses are upregulated [62].

To elucidate this positive point of return, it should be reminded that defective tryptophan catabolism contributes to excessive inflammation in CGD individuals infected with *Aspergillus fumigatus*. In immunocompetent hosts, tryptophan metabolism regulates both antimicrobial resistance and tolerance to inflammation-inducing microbial stimuli. Since superoxide is a cofactor of IDO, which is the rate-limiting enzyme in tryptophan degradation, superoxide-induced activation of IDO is a central mechanism by which an equilibrium between antifungal resistance and immune tolerance is achieved. The enzyme IDO represents both the host's effector defense mechanism and a means of properly balancing the generation of *inflammatory* Th17 and *regulatory* T cells (T_regs_) [63]. T_regs_ and IL-17-producing Th17 cells call forth opposing responses in inflammation, including aspergillosis. Whenever harmful inflammation predominates, whether microbial or sterile in origin, this IL-17-driven inflammation is associated with a decrease in anti-inflammatory T_reg_ cellular responses [64]. Basically, the same holds true for invasive aspergillosis as well as for human ischemia-reperfusion injury, no matter which organ is affected [65]. In all these cases, an attempt at a defense reaction translates into a control-evading detrimental inflammation. In terms of cellular adaptive immunity, expression of mRNA for transcription factors Tbet/Foxp3, which is indicative of Th1/T_reg_ activation, is increased in CD4^+^ T cells from wild-type, *disease-resistant* mice. Conversely, ROR*γ*t/Gata3 transcription factor mRNA expression, which is indicative of the opposite Th17/Th2 activation, is increased in CD4^+^ T cells from *disease-prone* p47phox^−/−^ mice. Early administration of PTX3 to p47phox^−/−^ mice restores this malevolent balance in that it reverses the original ratio of the transcription factors: it *increases* the expression of Tbet/Foxp3 and *decreases* that of ROR*γ*t/Gata3 [66]. It is tempting to speculate that comparable effects of PTX3 would be operative in patients with myocardial infarction, but reliable proofs thereof are as yet lacking.

## 13. Acquired Cellular Immunity in Inflammation

It is well established that CD4^+^T cells take part in the pathogenesis of atherosclerosis [67]. In addition to classical T_h_1 and T_h_2 helper cellular subsets, each producing its own set of cytokines which are mutually antagonistic, a third subset has been described recently, namely, the Th17 cells [68, 69]. These cells express proinflammatory cytokines of their own. The most important of them has been named interleukin- (IL-)17. Actually, IL-17 is a common denomination for a set of closely related cytokines referred to as IL-17A to IL-17F, respectively. From the clinical point of view, IL-17A and IL-17E appear to be the most active ones [70]. The orphan nuclear receptor *retinoic acid-related orphan receptor *γ*t* (ROR*γ*t) has been proposed as the key transcription factor for the differentiation of the Th17 lineage [71]. Th17 cells have been shown to play a role in chronic inflammation which, historically, was attributed to Th1 cells. One of these conditions is atherosclerosis [72]. Recent studies found that Th17 cells and their effector cytokine IL-17 are really increased in peripheral blood of patients with atherosclerosis, including acute coronary syndromes [73, 74]. Pathophysiologically, the Th17/IL-17^+^ cells directly support the development of atherosclerosis in ApoE^−/−^ mice. Herein, the amount of Th17 cells and of ROR*γ*t in affected vessels is proportional to the magnitude of the overall atherosclerotic burden. Both mediators are not manufactured *in situ, *as can be inferred by their respective behavior in ApoE^−/−^ mice. The number of Th17 cells in spleens of young, not yet atherosclerotic ApoE^−/−^ mice, has been proved comparable to that in healthy control animals. In ApoE^−/−^ mice, the number of Th17 cells tended to increase as a function of time, tightly paralleling continuing development of atherosclerosis. Although CD4^+^ T cells are the main source of IL-17, they are not the only origin of this cytokine. Neutrophils, as well as the CD8^+^ and *γδ* ^+^ T cells, were demonstrated to generate IL-17 in response to the inflammatory cytokine IL-15, just to give an example. Differentiation of mature Th17 cells depends on the *stimulation* with a “cocktail” of cytokines, namely, IL-6, TGF-*β*, and IL-21 and on the *induction *of their inherent transcription factor ROR*γ*t. On the other hand, IL-23 is indispensable to the achievement of mature Th17 phenotype and its stability under inflammatory conditions [75, 76].

The Th17/T_reg_ equilibrium is vital to the control of inflammation [77, 78]. Its derangement will bring about destabilization of atherosclerotic plaques, the composition of which has been extended recently to include Th17 cells, the cytokine IL-17A, and the transcription factor ROR*γ*t. If the fragile Th17/T_reg_ balance is lost, its new inadvertent ratio will call forth the onset of acute coronary syndromes (instable angia pectoris or acute myocardial infarction). In this context, patients experience significant increase in peripheral blood Th17 cell numbers, Th17-related cytokines (IL-17A, IL-6, IL-23) and ROR*γ*t levels. At the same time, there is a decrease in blood levels of T_reg_ numbers and T_reg_-related cytokines IL-10 and TGF*β* as well as of their transcription factor FoxP3. It may be soundly hypothesized that PTX3, which is endowed with the capacity to reverse this harmful inflammatory dysbalance in *Aspergillus fumigatus *infection by skewing the predominance of transcription factors (ROR*γ*t/Gata3 → Tbet/Foxp3) with consequent shift of their respective cellular subsets (Th17/Th2 → Th1/T_reg_), might bring about a comparable beneficial effects in this form of sterile inflammation. Nevertheless, it must be strongly pointed out that results yielded by experimental animals cannot by dogmatically extrapolated to humans, as attractive as they may appear [79].

## 14. Concluding Remarks

In both acute infections and acute coronary syndromes, the role of PTX3 appears to be primarily protective in that the long pentraxin dampens the inappropriate, exaggerated inflammatory response. On the local level, PTX3 released by PMNs or produced by monocytes/macrophages will abolish neutrophil recruitment and oxidative burst, a condition which kills bacteria by reactive oxygen species, while it inflicts collateral damage to the host. PTX3 co-operation with the adaptive immune system in defense inflammation which combats infection supports, in its turn, development of a protective Th1/T_reg_ immune response while at the same time it limits harmful inflammation elicited by the Th17/Th2 immune response. Further studies are needed in this context to furnish data that would imply to which extent PTX3 might be employed therapeutically.
